# Bone metastases from differentiated thyroid carcinoma: heterogenous tumor response to radioactive Iodine therapy and overall survival

**DOI:** 10.1007/s00259-022-05697-w

**Published:** 2022-02-12

**Authors:** Arnaud Jannin, Livia Lamartina, Coralie Moutarde, Mehdi Djennaoui, George Lion, Benjamin Chevalier, Marie Christine Vantyghem, Frédéric Deschamps, Julien Hadoux, Eric Baudin, Martin Schlumberger, Sophie Leboulleux, Christine Do Cao

**Affiliations:** 1 Department of Endocrinology and Metabolism, University Hospital of Lille, Lille, France; 2 University of Lille, Lille, France; 3 Gustave Roussy, Service d’oncologie Endocrinienne, Département d’Imagerie, University Paris Saclay, Cedex Villejuif, France; 4 Department of Endocrinology and Metabolism, Armentières Hospital, Armentières, France; 5 Department of Public Health, Valenciennes Hospital, Valenciennes, France; 6 Department of Nuclear Medicine, University Hospital of Lille and Lille University, Lille, France; 7 Gustave Roussy, Department of Interventional Radiology, University Paris Saclay, Villejuif Cedex, France

**Keywords:** Bone metastases, Thyroid carcinoma, Focal treatment, Radioiodine, Skeletal-related events

## Abstract

**Purpose:**

Bone metastases (BM) from differentiated thyroid carcinoma (DTC) impact negatively the quality of life and the life expectancy of patients. The aim of the study was (a) to evaluate the overall survival (OS) and prognostic factors of OS and (b) to assess predictive factors of complete BM response (C-BM-R) using radioiodine treatment (RAI) either alone or in association with focal treatment modalities.

**Methods:**

A total of 178 consecutive DTC patients harbouring BM, treated between 1989 and 2015, were enrolled in this retrospective study conducted in two tertiary referral centers. OS analysis was performed for the whole cohort, and only the 145 considered non-RAI refractory patients at BM diagnosis were evaluated for C-BM-R following RAI.

**Results:**

The median OS from BM diagnosis was 57 months (IQR: 24–93). In multivariate analysis, OS was significantly reduced in the case of T4 stage, ^18^FDG uptake by the BM and RAI refractory status. Among the 145 DTC considered non-RAI refractory patients at BM diagnosis, 46 patients (31.7%) achieved a C-BM-R following RAI treatment, either alone in 32 (18%) patients or in association with focal BM treatment modalities in 14. The absence of extra-skeletal distant metastasis and of ^18^FDG uptake in BM were predictive for C-BM-R.

**Conclusions:**

In nearly one-third of DTC patients with RAI avid BM, RAI alone or in combination with BM focal treatment can induce C-BM-R. The presence of ^18^FDG uptake in BM is associated with an absence of C-BM-R and with a poor OS. ^18^FDG PET-CT should be performed when BM is suspected.

**Supplementary Information:**

The online version contains supplementary material available at 10.1007/s00259-022-05697-w.

## Introduction

The incidence of DTC patients with distant metastases is estimated between 6 and 7 per million population each year [1–7]. Distant metastases are the leading cause of DTC-related morbidity and death [4, 5, 8, 9]. The most common sites of DTC metastases are lungs and bones, followed by brain and liver [6, 10].

Most bone metastases (BM) develop during follow-up and sometimes after a disease-free interval of years and frequently in association with lung metastases [3, 6, 10–13]. Some DTC histotypes, such as follicular and Hurthle cell thyroid cancers, present frequently with BM [14]. The most common sites of BM are spine (34.6%), pelvis (25.5%), sternum and ribs (18.3%), and extremities (10.2%) [15, 16]. The presence of BM often impairs the quality of life due to skeletal-related events (SRE), conventionally defined as the occurrence of pain, pathological fracture, spinal cord compression, hypercalcemia, or need for focal treatments, including percutaneous thermal ablation, cementoplasty, external beam radiation therapy (EBRT), or surgery [12, 17, 18]; the occurrence of a SRE is associated with a worse overall prognosis [3, 11, 12].

BM are usually tied with advanced DTC with poor prognosis, but they have been poorly investigated in large cohorts of DTC patients at an early stage of their development, including the so-called “occult” asymptomatic BM that is only evidenced on RAI total body scan and not visible on cross-sectional imaging.

About two-thirds of DTC patients with distant metastases are not cured with RAI treatment [7]. RAI is usually considered less effective in BM treatment compared with lung or lymph node metastases, namely in the case of large BM lesions [19–21]. However, some reports, based on a limited number of patients, have shown that some BM can respond to RAI [16, 21–26].

To improve our knowledge on BM management, we conducted a retrospective analysis of a large cohort of DTC patients with BM who were followed since their primary treatment in 2 tertiary referral centers of the French national network TUTHYREF. We aimed at (a) characterize prognostic factors of overall survival (OS) and (b) to assess potential predictive factors of complete BM response (C-BM-R) using RAI treatment.

## Patients and methods

We retrospectively analyzed the electronic databases of two tertiary referral centers of the INCA-ENDOCAN TUTHYREF network: Lille University Hospital and Gustave Roussy (GR). Consecutive DTC patients treated from January 1989 to December 2015 and with a follow-up of ≥ 2 years were analysed; follow up data were collected until July, 30th 2020”.

Inclusion criteria were: histologically confirmed DTC with BM, age ≥ 18 years, treatment with total thyroidectomy and RAI and complete follow-up data for ≥ 2 years from BM diagnosis. Primary thyroid tumors were classified according to the WHO criteria valid on the date of diagnosis and grouped into the following categories: well-differentiated thyroid carcinoma (WDTC) including papillary thyroid carcinoma (PTC) and follicular thyroid carcinoma (FTC) (including Hürthle cell carcinoma); poorly differentiated thyroid carcinoma (PDTC) according to Turin consensus criteria or aggressive variants of differentiated thyroid carcinoma (including tall cell, columnar cell, diffuse sclerosing, and hobnail variants of papillary thyroid carcinoma). Patients with anaplastic or medullary thyroid carcinoma were excluded. Patients with DTC and BM who never received RAI treatment for any reason and those being already refractory to RAI at the time of BM diagnosis were included in the overall BM cohort but were excluded from the RAI therapeutic efficacy analysis cohort. The study was approved by the institutional review board of the two institutions. Informed consent was obtained from all alive patients included in the study.

BM were diagnosed radiologically and/or on biopsy and/or on the post-therapy RAI whole-body scan (WBS) images and/or on 18-fluorodeoxyglucose (^18^FDG) positron emission tomography (PET) computed tomography (CT). BM were considered RAI avid if RAI uptake was observed at the post-therapy WBS, FDG avid if FDG uptake was observed at any ^18^FDG PET-CT examination. Diagnosis of BM without RAI uptake was established by a tumour board review (through dynamic monitoring of imaging modalities and serial serum thyroglobulin [Tg] measurements) or confirmed on biopsy in atypical cases (for the exclusion of BM from another primary tumour).

Follow-up consisted of periodic physical examination, serum Tg and anti-Tg antibodies determinations obtained every 6–12 months on levothyroxine treatment and following TSH stimulation at the moment of each RAI treatment. Tg determination was considered not reliable in the presence of Tg antibodies. Morphologic assessment with one or more of the following techniques: bone scintigraphy, CT, magnetic resonance imaging (MRI) or ^18^FDG PET-CT was performed every 3 to 6 months according to a local tumor board decision.

BM were considered synchronous if discovered within 6 months following thyroid surgery and metachronous when they were discovered more than 6 months after thyroidectomy. The presence of extra-skeletal metastases was analyzed. SREs were defined as bone pain, hypercalcemia, pathological fracture, spinal cord compression, epiduritis, cauda equina syndrome or any situation requiring surgery, external beam radiation therapy (EBRT) or interventional radiology to prevent or treat the bone complication. BM appearance on cross-sectional imaging (osteolytic or osteoblastic vs no visible lesion) or on functional imaging at the time of discovery was recorded. According to CT appearance, BM were classified to be osteoblastic (BM Hounsfield unit (HU) value higher compared to surrounding bone tissue) or osteolytic (BM HU value lower than surrounding bone tissue). The number of BM was classified into 3 groups: single BM, 2 to 5, or more than 5 BM.

RAI treatment consisted of the administration of 3.7 GBq (100 mCi) or 5.5 GBq (150 mCi) of 131I after withdrawal of thyroid hormone treatment to obtain a TSH level ≥ 30 mUI/L. RAI diagnostic WBS was performed in the hypothyroid state, 6–12 months after thyroid ablation in patients followed up at Lille University Hospital.

The therapeutic response of BM was defined after a centralized review of images and reports according to the Response Evaluation Criteria in Solid Tumors version 1.1 (RECIST 1.1) for all cases when BM were measurable.

In the absence of extra-skeletal metastases:

### Complete-BM-Response, C-BM-R

Disappearance of any RAI uptake in BM on WBS, disappearance and absence of FDG uptake of BM or disappearance of BM on morphological examinations and without the appearance of new BM, associated with undetectable Tg (< 1 ng/ml on l thyroxine treatment in the absence of Tg antibodies).

### Stable disease

Decrease or stabilization of RAI uptake without appearance of the new uptake foci associated with the absence of progression of any morphological BM and stabilization or non-significant increase in Tg (< 50% increase at 6 months after RAI administration).

### Progressive disease

Increase in the number and/or extent of the BM according to the RECIST 1.1 after one or more RAI therapeutic administrations and/or ^18^FDG PET-CT metabolic progression and/or an increase of the Tg level [27].

In the presence of extra-skeletal metastases or when the tumour response was discordant between BM and other lesions (when some lesions grew and others regressed), only morphologic and metabolic BM response to treatment was considered, regardless of the extra-BM response or Tg variation.

Some patients required additional focal treatments for BM such as EBRT, thermal ablation (radiofrequency or cryotherapy), cementoplasty, or surgery to achieve a therapeutic response. In both centers, decision-making involved a multi-disciplinary local board dedicated to the management of bone metastases to assist the clinicians in treatment choices between local and/or systemic treatment options depending on BM clinical presentation and the assessment of the individual risk of complications. Bone antiresorptive agents prescribed in case of the osteolytic lesion were bisphosphonates or denosumab, a RANK ligand inhibitor approved in France since 2011.

Patients were considered RAI–refractory at diagnosis or during follow-up according to the following criteria [7]: at least one measurable lesion without RAI uptake on any iodine-131 scintigraphy, or at least one measurable lesion that had progressed according to RECIST version 1.1 within 12 months after RAI therapy despite iodine-131 avidity at the time of treatment.

### Statistical analysis

Categorical data are expressed as numbers and percentages, continuous variables are expressed as means (standard deviation). Predictive factors associated with RAI response were analyzed using the Wilcoxon test (continuous variables) and the chi-square test or Fisher exact test (categorical variables) for univariate analysis and logistic regression for multivariate analysis. OS predictive factors were analyzed using Log-rank test for univariate analysis and Cox proportional hazards model for multivariate analysis. A *p*-value < 0.05 was considered statistically significant for all analyses. Analyses were performed with SPSS Statistics v24.0.1 (IBM Corp, Armock, NY) and R v4.0.0 (R Foundation for Statistical Computing, Vienna, Austria).

## Results

### Patient characteristics at the time of BM diagnosis

**Fig. 1:**
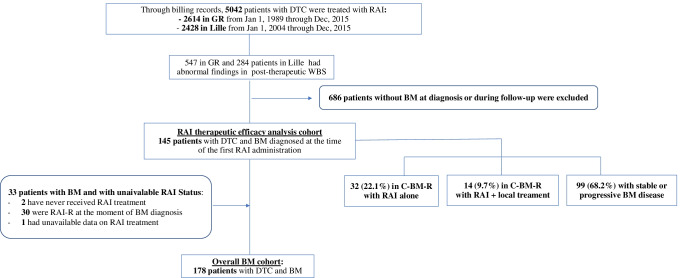
Flowchart. DTC: Differentiated thyroid carcinomar, GR: Gustave-Roussy, BM: Bone metastases, RAI: radioactive iodine, RAI-R: Radioactive iodine Refractory, C-BM-R: Complete bone metastases response

From 1989 to 2015, 2614 DTC patients were treated with RAI in GR, and from 2004 to 2015, 2224 DTC patients were treated with RAI in Lille (Fig. 1). One hundred and seven patients from GR and 71 patients from Lille for a total of 178 DTC patients had BM and formed the basis of this study (Fig. 1). Among these 178 DTC patients with BM, 60% were women with a median age of 59.5 (IQR: 48.2–67.7) years (Table 1). There were 85 (47,8%) WDTC, including 45 (25.3%) PTC and 40 (22.5%) FTC, 48 (27%) PDTC and 31 (17.4%) aggressive variants. Pathologic diagnoses were not available for 14 (7.8%) [28]. Fifty-seven of 136 patients who had a lymph node dissection (41.9%) at initial surgery had neck lymph node metastases, and 88 (49.4%) patients with BM had extra-skeletal distant metastases at the diagnosis of BM. Among the 171 patients with available information on BM sites, 86 had spinal BM (50.3%), 57 had pelvic BM (33.3%), 50 had BM located in sternum and ribs (29.2%), 35 had BM in extremities (20.4%), 25 in shoulder girdle (14.6%) and 20 in the skull (11.7%). Single BM occurred in 63 (35.4%) patients involving spine (17/63, 27%), pelvis (13/63, 20.6%), ribs and the sternum (11/63, 17.5%), extremities (9/63, 14.3%), skull (7/63, 11.1%), and shoulder girdle (6/63, 9.5%).

**Table 1:** Characteristics of patients with bone metastasis from differentiated thyroid carcinoma

Characteristics (*n* = 178)	Number of patients (%)
Sex	
Male	72 (40.4%)
Female	106 (59.6%)
Age at diagnosis of DTC with BM (years) – median [range]	59.5 [48.2–67.7]
Time DTC-BM diagnosis (months) – median [range]	60 [24–96]
Cancer type/histology	
WDTC	85 (45.8%)
PTC	45 (25.3%)
FTC	40(22.5%)
PDTC	48 (27%)
Aggressive variants*	31 (17.4%)
Unknown	14 (7.9%)
Necrosis	
Presence	55 (30.9%)
Absence	81 (45.5%)
Unknown	42 (23.6%)
Vascular invasion	
Presence	106 (59.6%)
Absence	51 (28.6%)
Unknown	21 (11.8%)
TNM T Staging at diagnosis	
T1	23 (12.9%)
T2	46 (25.8%)
T3	77 (43.3%)
T4	26 (14.6%)
Unknown	6 (3.4%)
Presence of metastatic lymph nodes at diagnosis	57 (41.9%)^1^
Presence of extra-skeletal metastasis at diagnosis	88 (49.4%)
Number of BM sites	
1	63 (35.4%)
2–5	75 (42.1%)
> 5	40 (22.5%)
Synchronous BM	112 (62.9%)
Evidence of BM on cross-sectional imaging	133 (74.7%)
Presence FDG PET/CT uptake in BM	
Yes	100 (56.2%)
No	33 (18.5%)
Unknown	45 (25.3%)
SUVmax—median [range]	6.1 [4.5–12.8]^2^
BM size (cm)—median [range]	3 [2-6]^3^
Stimulated Tg at the first RAI—median [range] (ng/ml)	193.4 [16.6–3200]

*DTC*, differentiated thyroid carcinoma; *BM*, bone metastases; *PTC*, papillary thyroid carcinoma; *FTC*, follicular thyroid carcinoma; *WDTC*, well-differentiated thyroid carcinoma; *PDTC*, poorly differentiated thyroid carcinoma; *TNM*, tumor node metastasis; *FDG PET*, fluoro-desoxyglucose-positron emission tomography; *SUV*, standardized uptake value; *Tg*, thyroglobulin; *RAI*, radioactive iodine; *SRE*, skeletal-related events. ^1^Neck dissection not performed in 42 patients, ^2^Unknown for 106 patients, ^3^Unknown for 106 patients. *aggressive variants: tall cell, columnar cell, hobnail, or diffuse sclerosing variants

More than half (62.9%) of the patients had synchronous BM. BM-diagnosis was clinical (accounting for a SRE) in 42.1% of the patients. In the other 68%, BM were detected on post-therapeutic WBS (*n* = 74) or on cross-sectional or ^18^FDG PET-CT imaging (*n* = 29). Synchronous BM presentation was strongly associated with RAI uptake of BM (*p* = 3.83 × 10^−13^). In patients with metachronous BM, the median time from DTC diagnosis to BM was 60 months (IQR:24–96).

At the moment of their discovery, BM were multiple in 115 patients (66.6% of cases) and RAI uptake was present in all BM in 74 of these 115 patients (64.3%). BM were associated with structural abnormalities on CT-scan and/or MRI in 133 patients (74.7% of the cases) and were osteolytic in 116 (87.2%) patients and osteoblastic in 12 (9%) cases (unknown data for 5 patients).

^18^FDG PET-CT, performed in 133 patients, showed high FDG uptake in BM of 75.2% of explored patients. Among the 128 patients with available data on both ^18^FDG PET-CT and RAI uptake, 62 had both ^18^FDG and RAI uptake, 29 had RAI uptake without ^18^FDG uptake, 33 had ^18^FDG uptake without RAI uptake, and 4 patients had neither ^18^FDG nor RAI uptake.

The median value of TSH-stimulated Tg at the time of BM diagnosis was 175.5 ng/ml (IQR: 8.4–3750). Anti Tg antibodies were present in 23 patients.

### Treatment

Among the 178 patients with BM, 175 underwent RAI therapy (2 were directly treated with chemotherapy, and one had no available data). They received a median cumulative RAI activity of 300 mCi (IQR: 200–550 mCi) corresponding to a median number of 3 treatment courses (IQR: 2–5 courses). Of these 175 patients, 30 (17.1%) were classified as RAI refractory at the time of BM diagnosis and did not receive further RAI courses.

Focal treatment of BM was used in 123 patients (69.1%) consisting in surgery in 74 (41.6%), EBRT in 111 (63.4%), thermal ablation in 84 (47.5%) and cementoplasty in 72 patients (40.2%), and anti-resorptive agents in 49 patients (27.5%). A combination of several modalities was used in 54 patients. Other treatment modalities included cytotoxic chemotherapy in 14 patients (7.8%), tyrosine kinase inhibitors in 28 patients (15.7%), and pembrolizumab within a clinical trial in 5 patients (2.8%). Overall, 112 (62.9%) patients had a SRE during the study period. In the follow-up of these patients, nausea (20/175, 11.4%), salivary dysfunction (13/175, 7.4), and bone pain (11/175, 6.3%) were reported as acute effects of RAI therapy. Long-term effects of RAI therapy included sialadenitis (15/175, 8.6%) and leukemias (2/175, 1.2%). No RAI-induced solid cancer was noted.

### Prognostic factors of overall survival in the overall BM cohort

The median follow-up period was 72 months (IQR: 33–113) from the diagnosis of DTC. Eighty-one (45.5%) patients died during follow-up. The cause of death was thyroid carcinoma in 61 patients (75.3%), the other 20 deaths were not related to thyroid cancer. From DTC diagnosis, the median OS time was 81 months (IQR: 48.5–120), and the 3-year, 5-year, and 10-year OS rates were 82%, 70%, and 26%, respectively. From BM diagnosis, the median OS time was 57 months (IQR: 24–93) and the OS rates at 5 and 10 years from BM detection were of 56.5% and 15.3%, respectively. The OS rates at 5 and 10 years were 48.6 and 14.3%, respectively, for patients with initial BM RAI uptake but only 15.2 and 5.1%, respectively, for patients with no RAI uptake in BM. No center-effect and period-effect were observed (data not shown).

**Fig. 2:**
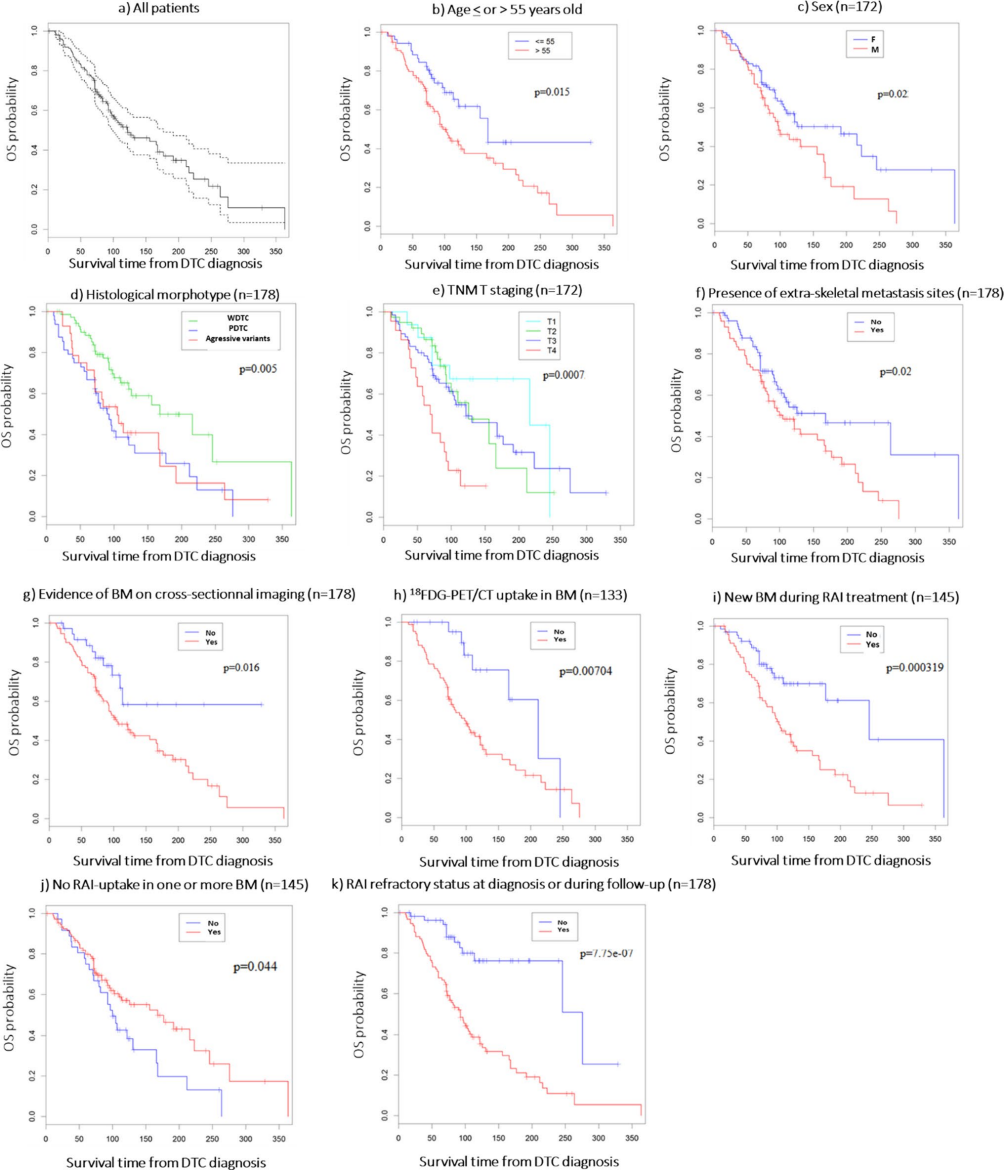
Overall survival univariate analysis of the overall BM cohort. Figure 2a: median OS of the overall BM cohort analysis with first or third interquartile range. 2b: OS analysis as a function ofage above or below 55 years. 2c: OS analysis depending on sex status ; 2d: OS analysis depending on histologicalmorphotype ; 2e: OS analysis depending on TNM T staging ; 2f: OS analysis depending on the presence of extra-skeletalmetastasis sites ; 2g: OS analysis as function of BM on cross-sectional imaging ; 2h: OS analysis depending on 18FDG-PET/CTuptake in BM ; 2i: OS analysis depending on new BM during RAI treatment ; 2j: OS analysis depending on the absence of RAIuptakein one or more BM ; 2k: OS analysis depending on the refractory status at diagnosis or during follow-up.The *p* values are presented for Log-rank tests.OS: Overall survival, DTC: Differentiated Thyroid Carcinoma, WDTC: Well-Differentiated Thyroid Carcinoma, PDTC: PoorlyDifferentiated Thyroid Carcinoma, BM: Bone metastasis, TNM: Tumor Node Metastasis. 18FDG PET-CT: 18-Fluorodesoxyglucose-positron emission tomography, RAI: radioactive iodine.

**Fig. 3:**
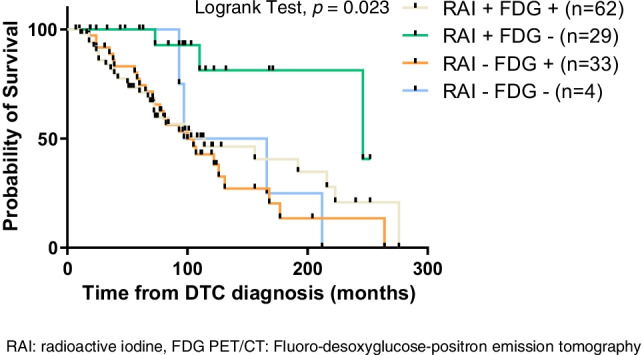
Kaplan–Meier graph of overall survival in 178 patients with differentiated thyroid carcinomas and bone metastases depending on RAI and/or FDG-PET/CT uptake. RAI: radioactive iodine, FDG PET/CT: Fluoro-desoxyglucose-positron emission tomography

In the univariate analysis, a poor OS was significantly associated with male sex (*p* = 0.02), age > 55 years at the time of the diagnosis of BM (*p* = 0.015), PDTC and aggressive histological variants (*p* = 0.005), T4 stage (*p* = 0.0007) at diagnosis, presence of extra-skeletal metastatic sites (*p* = 0.02), absence of RAI uptake in one or more BM (*p* = 0.044), presence of BM visible on cross-sectional imaging (*p* = 0.02), ^18^FDG PET-CT uptake at the sites of BM (*p* = 0.007), RAI refractory status at diagnosis and during follow-up (*p* = 0.0000007) or discovery of new BM during RAI treatment (*p* = 0.0003) (Fig. 2). RAI- uptake without ^18^FDG PET-CT uptake in BM is associated with a longer OS (median 95 months (IQR: 48–124.5)) (*p* = 0.026) (Fig. 3). The absence of focal treatment was associated with a longer OS (*p* = 0.0114), and no OS differences were observed between the type of local treatment (data not shown). No difference in OS was observed between patients receiving local treatment before (33/123 patients) or after a SRE (89/123 patients) (*p* = 0.3).

**Fig. 4:**
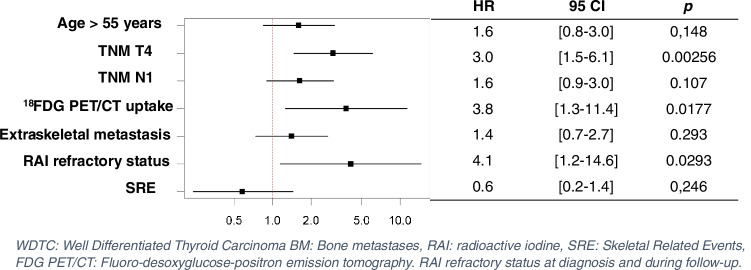
Forest plot of multivariate-adjusted hazard ratios (HRs) for factors related to mortality outcome in DTC patients with BM. WDTC: Well Differentiated Thyroid Carcinoma BM: Bone metastases, RAI: radioactive iodine, SRE: Skeletal Related Events, FDG PET/CT: Fluoro-desoxyglucose-positron emission tomography. RAI refractory status at diagnosis and during follow-up

At multivariate analysis, including 7 most significant variables in relation to the number of deaths, OS was significantly lower in patients with a T4 thyroid tumor, ^18^FDG uptake at BM sites and RAI refractory status at diagnosis and during follow-up (*p* = 0.00256, Hazard-ratio (HR) = 3.0; 95% CI, 1.5 to 6.1; *p* = 0.0177, HR = 3.8; 95% CI 1.3 to 11.4; *p* = 0.0293, HR = 4.1; 95% CI 1.2 to 14.6) (Fig. 4).

### Predictive factors associated with success of RAI alone or in combination to locoregional treatment for BM

Among the 178 patients included, 145 patients treated with RAI after the diagnosis of BM were included in the RAI therapeutic efficacy analysis cohort (Fig. 1). The other 33 patients were excluded from this cohort: 2 patients had never received RAI treatment, 30 had no detectable RAI uptake in the BM at the time of BM diagnosis and were considered iodine refractory, and data were unavailable in one. The median OS from BM diagnosis was 70 months (IQR: 35–100) for the 145 patients treated with RAI and 24 months (IQR:13–45) for the other 33 patients.

At the time of the last RAI treatment of the 145, RAI treated patients, 46 patients (31.7%) achieved a C-BM-R, 61 (42.1%) had stable disease, and 38 (26.2%) had progressive disease. In the 46 patients who achieved C-BM-R, OS was 84% at 5 years and 29.8% at 10 years, a figure that is significantly higher (*p* < 0.001) than in patients who did not achieve C-BM-R (64.1% at 5 years and 14% at 10 years).

Among the 46 patients who achieved a C-BM-R, pathology was WDTC in 40 and PDTC or aggressive variants in 6. The median Tg level following TSH stimulation at the time of the discovery of BM for a patient with C-BM-R was relatively low at 12.5 ng/ml (IQR: 3.9–31.1) in accordance with the low tumor burden and was 0.7 ng/ml (IQR: 0.07–8) after RAI treatment. FDG PET/CT was performed in 28, and 8 patients had a BM ^18^FDG uptake, and 20 did not. The median cumulative RAI activity administered before C-BM-R was 200 mCi (IQR: 200–375). Thirty-four patients (73.9%) with C-BM-R had received a cumulative RAI activity < 350 mCi (12.9 GBq). In 32 cases, C-BM-R was reached with RAI alone, whereas in 14 cases, focal treatments were applied in one or more BM sites (Table 2), and five were treated with a bone anti-resorptive agent. A dissociated response with the coexistence of C-BM-R and extraskeletal non-responding lesions after RAI treatment was observed in 4 of these 46 cases (8.7%). Of these 46 patients, 26 (56%) had BM without any structural abnormality on cross-sectional imaging, 22 (47.8%) had only one BM, and only 11 (26%) patients had extra-skeletal distant metastases. Twenty-three patients of 26 (88.5%) with BM without structural abnormality on cross-sectional imaging reached a C-BM-R with RAI alone. Nine patients among the 20 (45%) with BM with structural abnormality reached C-BM-R with both RAI and focal treatments.

**Table 2:** Predictive factors associated to the absence of complete BM response in the 145 patients of the RAI therapeutic efficacy analysis cohort

	Complete BM-response			Univariate †	Multivariate††	OR
Variables	No (*n* = 99)		Yes (*n* = 46)	*p*	*p*	95% CI
Prognostic factors available at the moment of first RAI course						
Sex (male)	44 (81.5%)	10 (18.5%)		0.00849		
Age (years)*	62 [53–68]	46 [31–65]		0.000224		
Age > 55 years	68 (81.9%)	15 (18.1%)		0.0000437	0.157	2.8 [0.7–10]
TNM T stage						
T1	12 (11%)	10 (22%)		0.24		
T2	26 (27%)	15 (33%)				
T3	43 (45%)	16 (36%)				
T4	16 (17%)	4 (9%)				
TNM N1 stage	34 (45%)	10 (28%)		0.0946		
Histology/cancer type				0.00000629	0.54	0.4 [0.1–2]
WDTC	47 (43%)	40 (85%)				
PTC	14 (14.1%)	27 (27.3%)		0.000436		
FTC	33 (33.3%)	13 (13.1%)				
PDTC	31 (34%)	4 (11%)				
Aggressive variants	21 (23%)	2 (4%)				
Necrosis	30 (46%)	5 (12%)		0.00142		
Vascular invasion	59 (72%)	20 (47%)		0.01721		
Synchronous BM	70 (61%)	39 (83%)		0.0679		
Time DTC-BM diagnosis *	0 [0;3]	0 [0;0]		0.007		
Extra-skeletal metastasis	**56 (56%)**	**11 (26%)**		**0.000242**	**0.0052**	**9.1 [2.2–50]**
SRE at diagnosis	52 (46%)	34 (28%)		0.0147		
High-risk fracture	76 (77%)	27 (60%)		0.0256		
Number of BM sites				0.332		
1	31 (32%)	20 (43%)				
2–5	45 (45%)	16 (36%)				
> 5	23 (23%)	10 (21%)				
RAI uptake in one or more BM	82 (73%)	44 (96%)		0.0332		
RAI uptake in all BM	69 (65%)	39 (93%)		0.00954	0.062	0.1 [0.01–1.7]
Evidence of BM on cross sectional imaging	80 (83%)	20 (43.5%)		0.00000613	0.598	1.5 [0.3–6.3]
BM size* (cm)	3.4 [2-6]	3 [2.3–4.67]		0.25		
Osteolysis	66 (79%)	13 (42%)		0.000309		
FDG PET/CT uptake in BM	**67 (85%)**	**8 (35%)**		**0.000000575**	**0.0236**	**5 [1.3–25]**
Tg at diagnosis (ng/ml)**	1325 [107.5–6502.5]	12.5 [3.9–31.1]		0.000000796		
Factors available during follow-up						
Median follow-up time (years) [range]	7 [4–10.2]	7 [4–10.5]		0.855		
RAI number of cures*	3 [1-5]	2 [2, 3]		0.00254		
Cumulative RAI activity*	400 [200–600]	200 [200–375]		0.00209		
RAI Refractory during follow-up	70 (70.7%)	3 (6.5%)		6.28*10^^−^13		
New BM during treatment	55 (55.6%)	5 (10.8%)		3.56*10^^−^08		
Occurrence of SRE	73 (73.7%)	11 (23.9%)		1.55*10^^–^08		
Numbers of SRE per patient*	2 [1-3]	0 [0–1]		3.3*10^^−^9		
Anti-resorptive agents	32 (32.3%)	6 (13%)		0.0151		
BM surgery	53 (53.5%)	8 (17.4%)		0.0000408		
Radiotherapy or Thermal ablation	72 (72.7%)	9 (19.6%)		1.97*10^^−^9		
Cementoplasty	57 (57.6%)	10 (21.7%)		0.0000439		
Systemic treatment				0.00005		
None	72 (72.7%)	46 (100%)				
Chemotherapy	10 (10.1%)	0				
TKI	17 (17.3%)	0				
All-cause deaths	54 (54.5%)	5 (10.8%)		4.18*10^^−^07		

*OR*, odds ratio; *TNM*, tumor node metastasis; *WDTC*, well-differentiated thyroid carcinoma; *PDTC*, poorly differentiated thyroid carcinoma; *PTC*, papillary thyroid carcinoma; *FTC*, follicular thyroid carcinoma; *BM*, bone metastases; *DTC*, differentiated thyroid carcinoma; *RAI*, radioactive iodine; *FDG PET*, fluorodesoxyglucose-positron emission tomography; *RAI*, radioactive iodine; *SRE*, skeletal-related events; *Tg*, thyroglobulin; *TKI*, tyrosine kinase inhibitor. *mean and standard deviation. **median and range. †Wilcoxon test/chi-square test or Fisher exact test. †† Logistic regression

Six of the 46 patients (13%) who achieved a C-BM-R experienced a subsequent recurrence with a median time to recurrence of 36 months (IQR: 19–47). Three patients had a bone recurrence in other bone sites, 3 had an extraskeletal recurrence, and 4 became then refractory to RAI. Five had BM ^18^FDG PET-CT uptake, and four had extraskeletal metastases at the time of BM diagnosis.

**Fig. 5:**
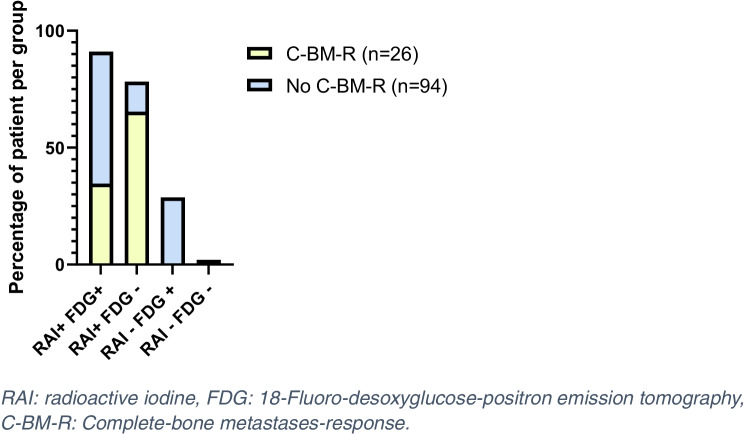
Radioactive iodine and 18-FDG-uptake in bone metastases of patients with (*n* = 26) or without (*n* = 94) complete bone metastases responses. RAI: radioactive iodine, FDG: 18-Fluoro-desoxyglucose-positron emission tomography, C-BM-R: Complete-bone metastases-response.

Focusing on the 99 patients who did not achieve C-BM-R with RAI, pathology was WDTC in 48 and PDTC or aggressive variants in 51 patients. Fifty-six (56%) patients had extra-skeletal metastasis. The median cumulative administered RAI activity was 400 mCi (IQR: 200–600). ^18^FDG PET/CT has performed in 80 of these 99 patients, and 67 patients had a BM ^18^FDG uptake, and 13 did not (Fig. 5).

Table 2 shows the univariate and multivariate analysis of the predictive factors for the absence of C-BM-R in these 145 patients who were treated with RAI. In the multivariate analysis, the presence of extra-skeletal distant metastasis and the presence of ^18^FDG uptake were predictive for the absence of C-BM-R (Table 2). Among the 105 patients who had a ^18^FDG PET/CT, patients without ^18^FDG uptake in BM had a C-BM-R rate of 16.2% (17/105) and this rate was only 8.5% (9/105) in those with FDG uptake. Patients without extra-skeletal distant metastasis had a C-BM-R of 24.1% (35/145) and this rate was only 7.6% (11/145) in those with extra-skeletal distant metastasis. Patients without ^18^FDG uptake in BM and without extra-skeletal distant metastasis had a C-BM-R of 71.4% (15/21) and this rate was only 9.3% (4/43) in patients with ^18^FDG uptake in BM and with extra-skeletal distant metastasis.

When considering patients with RAI uptake in all BM and harboring no ^18^FDG uptake C-BM-R reached 84.7% (39/46). Among the 17 patients with C-BM-R without ^18^ FDG uptake in BM, local treatments were used in only 4 patients; whereas, among the 9 patients with C-BM-R and ^18^FDG uptake in BM, 7 needed local treatments and 2 were treated with RAI alone (these 2 patients had RAI and ^18^FDG uptake in BM). The 42 patients with RAI-avid BM and without any structural abnormality on cross-sectional imaging had a 61.9% (26/42) rate of C-BM-R (Table 2), compared to a 21.3% (19/89) C-BM-R rate for a patient with RAI-avid BM and BM visible at cross-sectional imaging.

## Discussion

In DTC patients, the presence of BM is associated with a decreased survival rate [3, 10, 11, 23, 29] and with impairment of quality of life due to SRE [12, 17, 30]. We had the opportunity to analyze the data obtained from a comprehensive series of DTC patients managed from the early detection of BM in two expert centers.

We found that nearly one-third of DTC patients with RAI avid BM can achieve C-BM-R with RAI treatment alone or in combination with BM focal treatment. We also confirm the negative prognostic impact of ^18^FDG uptake in BM for OS and complete BM response.

Limitations of our study are the diversity of BM management due to its retrospective design, some missing information, the long-time span of accrual (1989 to 2015) with differences in pathological classification of the tumors, imaging modalities used for the detection and follow-up of BM, and in treatment options, such as TKIs and focal treatment modalities. Nevertheless, the sample size analyzed was large, the median follow-up was long, and the patients were followed in referral centers with therapeutic proposals routinely decided by multidisciplinary local tumor boards.

First, we observed a median OS of 57 months (IQR: 24–93) from BM diagnosis with an OS rate of 56.5% at 5 years and of 15.3% at 10 years. These results are in accordance with previous survival rates of DTC patients with BM, with a 5-year OS rate of 58.7–65% [31, 32] and a 10-year OS rate of 13–21% [5, 23, 31, 33, 34]. Our survival data are also quite similar to those reported in studies of DTC patients with lung metastases [21, 35]. In the multivariate analysis, OS was significantly lower in the case of T4 tumor, ^18^FDG PET-CT uptake, and RAI refractory status. Thus, it appears that functional imaging provides an overview of the biological aggressiveness of all metastatic lesions. T4 TNM stage was reported as an independent prognostic factor for poor OS in DTC patients with BM, and this is also consistent with recent studies in metastatic DTC patients [36–39]. Other prognostic factors, such as age and SRE, previously shown as pejorative risk factors, and radiotherapy, previously shown as a protective factor for SRE, were not confirmed as independent risk factors in our study [1, 11, 12, 16, 31, 40]. A worse OS was found in the patients needing focal treatments, likely because these more heavily treated patients are also those with a more advanced and aggressive RAI-refractory disease.

Indeed, OS of metastatic DTC patients is significantly better in patients with RAI-avid distant metastases compared to those with RAI-non-avid distant metastases [1, 5, 11, 21, 25, 41–43]. In DTC, as in other cancers, tumor heterogeneity is a major issue, and ^18^FDG PET-CT may indicate both the degree of dedifferentiation of metastatic tumor cells and their aggressiveness. Indeed, there is an inverse relationship between RAI and ^18^FDG accumulation in DTC cells [44]. ^18^FDG uptake also signals aggressive disease, and we confirm that patients with RAI-avid and ^18^FDG non-avid distant metastasis have a better prognosis [45]. Patients with both ^18^FDG and RAI uptake in the same lesion or in different lesions represent a heterogeneous group of patients, but their prognosis seems to be similar to the group with only ^18^FDG uptake [46]. However, some tumor responses to RAI treatment have been observed in patients with both RAI and FDG uptake, and such patients should indeed be treated with RAI [47].

Second, our study highlights that patients with iodine avid BM treated with RAI have a better prognosis and could achieve a C-BM-R in a substantial number of cases with the support of focal treatments. Responder patients are quite homogeneous regarding disease presentation and response, as the rate of dissociated response of BM vs other distant metastatic sites was 22% (15/67) in non-responders and only 8.7% (4/46) for the patients with C-BM-R. The rate of C-BM-R with RAI alone is 22.1% and is consistent with that reported by Durante but differs from the study by Lin in which only a 9.9% complete response rate was observed [6, 23]. This difference might be related to the higher rate of DTC with BM not visible at cross sectional imaging and the higher rate of RAI avid BM at diagnosis in our cohort. These BM are frequently asymptomatic and were mostly discovered on the post-therapy WBS. Indeed, nearly one-fourth of our population harbored iodine-avid BM that were not visible on cross-sectional imaging at first RAI treatment; these patients have a high C-BM-R rate (61.9%) compared with a 21.3% C-BM-R observed in patients who had RAI-avid BM and BM visible at cross-sectional imaging, in agreement with the previous results [48]. The absence of structural abnormality in BM was highly significant for C-BM-R at univariate but not at multivariate analysis, probably because it is closely related to both the absence of ^18^FDG uptake and of extra-skeletal lesions. In general, in the literature, RAI was less effective at treating BM vs lung metastatic sites, a finding that could be related to a higher RAI efficacy on small metastases and also on the distribution of RAI avid lesions. Indeed, patients with lung metastases -especially those with miliary presentation- showed higher remission rates (50–74%) than patients with larger lung metastases and those with multiple and large BM [6, 21]. We also found that BM without extra-skeletal metastases and absence of ^18^FDG PET-CT uptake are more frequently associated with a C-BM-R, making these patients ideal candidates for RAI. As already described in DTC patients with pulmonary metastases treated with RAI, the presence of metastasis at another site was also associated with a lower rate of C-BM-R, showing the impact of tumor burden on RAI overall response [21, 26, 35, 48]. Unfortunately, up to 13% of patients with C-BM-R had experienced DTC recurrence during subsequent follow-up, meaning that careful monitoring is needed even after complete remission.

The WDTC histological subtype compared with PDTC and aggressive variants were associated with C-BM-R in univariate analysis and not confirmed in multivariate analysis, suggesting that RAI treatment in presence of known BM should be carried out at least once, even in the presence of aggressive histotypes.

The median cumulative RAI activity in patients with C-BM-R was 200 (IQR: 200–375) vs 400 (IQR:200–600) in non-RAI responders*.* The question of appropriate modalities for RAI treatment for metastatic DTC patients is still pending: what is the optimal activity to be administered per treatment course, how many RAI treatment courses, and at which interval of time, which optimal cumulative I131 activity should be administered [49, 50]. There is no evidence for better efficacy in terms of OS of a dosimetric approach compared with an empirical approach for determining the treatment activity for DTC patients with distant metastasis [51]. This study confirms that the administration of fixed empirical activities of 3.7 GBq (100 mCi) or 5.5 GBq (150 mCi) after withdrawal of thyroid hormone treatment is effective. The safety of RAI treatment is now well established and confirmed in our study.

Though RAI uptake was present in all BM at diagnosis in more than two-thirds of our patients, 62.1% of these patients did not achieve a C-BM-R. Such discrepancies can be explained by different disease biology and different iodine uptake in metastatic foci where local concentration and retention of RAI may not be sufficient or heterogeneous to obtain a therapeutic effect. Also, the radiosensitivity of tumor cells may vary but is poorly investigated. Indeed, the ability of thyroid carcinoma tissue to concentrate RAI depends on the expression and functional integrity of the sodium-iodide symporter (*NIS*) and the iodine organification process [52]. It has been demonstrated that abnormal MAP-kinase pathway activation in tumor cells, due for instance, to a *BRAF* mutation, leads to downregulation of *NIS* expression. A recent study has estimated that about 20% of DTC with BM carry a mutation in *BRAF* and 42% in *RAS* genes, which could make them candidates for redifferentiation trials [31, 53, 54].

RAI alone did not permit to achieve a C-BM-R in some DTC patients who required additional focal treatment modalities (e.g., BM surgery, EBRT, or thermal ablation). Coupling both approaches have permitted to improve the C-BM-R rate by roughly 1/3. Indeed, C-BM-R was reached with RAI alone in 22.1% of cases and in 31.7% of cases treated with RAI and focal treatments applied to one or more BM sites. C-BM-R for patients with BM without structural abnormality in cross-sectional imaging was 88.5% with RAI alone and 45% for patients with BM with structural abnormality treated with RAI and focal treatment. The greater use of focal treatment modalities in our therapeutic strategies (69.1% in our study vs 51.9% in the literature) in oligo-metastases may have contributed to improving the response rate [23]. Considering that roughly 75% of C-BM-R were obtained with cumulative I131 activities lower than or equal to 350 mCi, indicating that response to RAI occurs early and that more than two-thirds of the patients had focal BM treatments, we might speculate that focal treatment could be more effective when performed early during the course of the disease. In addition, as suggested in our study, ^18^F-FDG PET/CT can also help to identify tumors with aggressive behavior that could benefit from the association of other therapeutic approaches such as focal treatments [55]. Finally, our results show that treatment combination with RAI alone or with focal treatments is effective to treat BM from DTC, which could allow delaying the initiation of systemic therapy such as TKIs known to cause more side effects and did not limit access to other systemic therapies in case of tumor progression. These results align with the most recent guidelines recommending the use of a multimodal approach consisting of anti-resorptive agents, EBRT, surgery, thermal ablation, cementoplasty, and TKI in patients with diffuse and/or symptomatic RAI refractory BM [56].

## Conclusion

This study confirms that almost one-third of DTC patients with BM (either single or multiple) can achieve a C-BM-R, especially those without extra-skeletal metastases, with RAI avidity in all BM lesions and without ^18^FDG PET-CT uptake. Applying focal treatments targeting BM in addition to RAI was highly effective in this context. ^18^FDG PET-CT is essential in the work-up of all DTC patients with BM to characterize BM behavior and extent, choose treatment modalities and determine prognosis. ^18^FDG PET-CT should be performed on a routine basis at the time of BM diagnosis, even if RAI uptake is present. Therapeutic strategies offered to DTC patients with BM should consider all relevant factors for specific outcome (RAI response, SRE, mortality) and be discussed within a dedicated multidisciplinary tumor board.

## Supplementary Information

Below is the link to the electronic supplementary material.

Supplementary file1 (PPTX 79 KB)
