# Supplementary material for: Bone metastases from differentiated thyroid carcinoma: heterogenous tumor response to radioactive Iodine therapy and overall survival
Source: Eur J Nucl Med Mol Imaging. 2022 Feb 12;49(7):2401–13. doi: 10.1007/s00259-022-05697-w (PMC9165254; doi:10.1007/s00259-022-05697-w)
Supplement: Supplementary file 1 — Supplementary file1 (PPTX 79 KB) [file 259_2022_5697_MOESM1_ESM.pptx]

## Slide 1
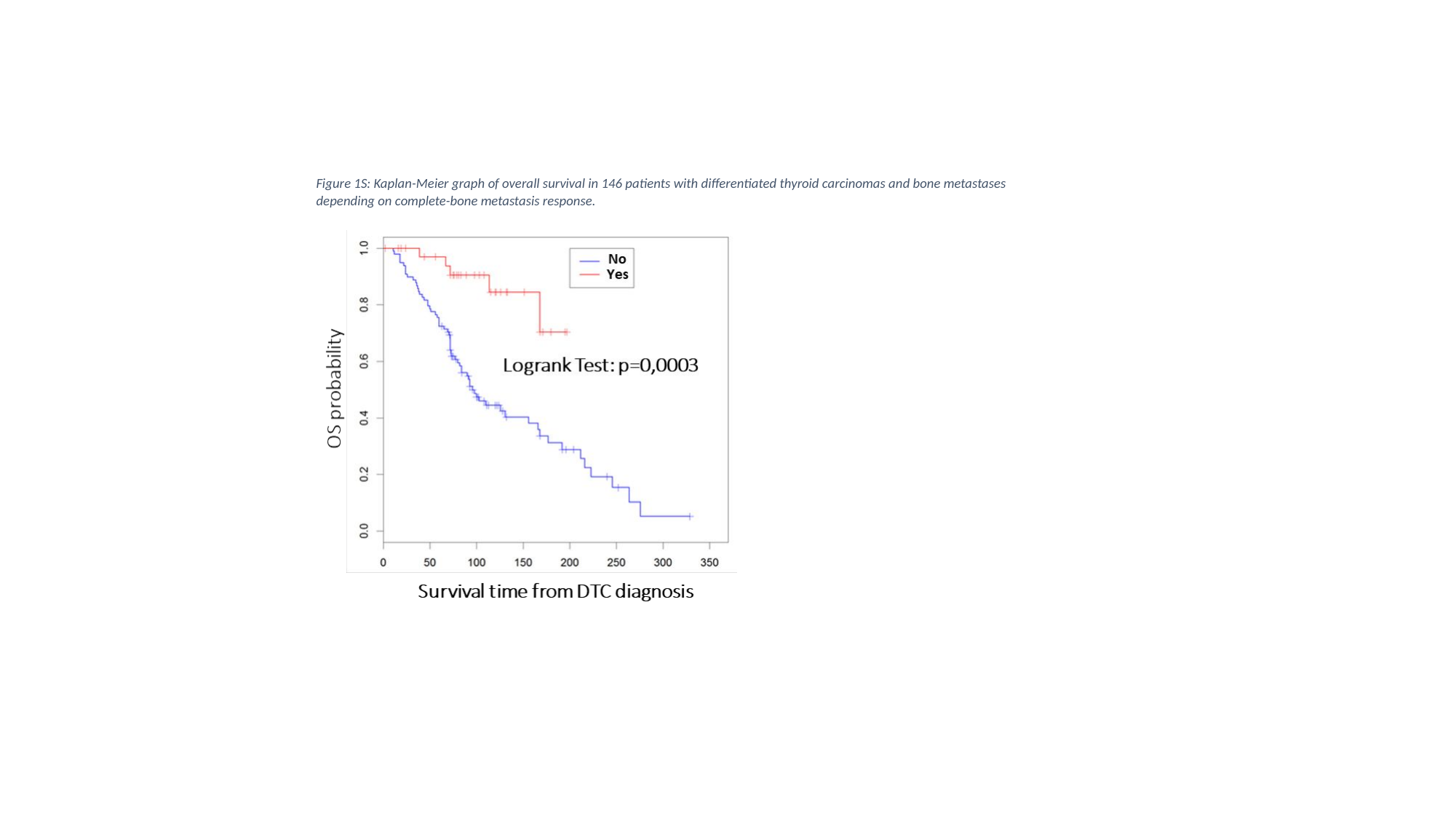

Figure 1S: Kaplan-Meier graph of overall survival in 146 patients with differentiated thyroid carcinomas and bone metastases depending on complete-bone metastasis response.
